# Serum Iron Status and Retinal Degenerative Diseases: A Mendelian Randomization Study on AMD, RP, and DR

**DOI:** 10.3390/nu16213747

**Published:** 2024-10-31

**Authors:** Sichang Qu, Yewen Zhu, Norbert Pfeiffer, Franz H. Grus

**Affiliations:** 1 Department of Experimental and Translational Ophthalmology, University Medical Center, Johannes Gutenberg University, 55131 Mainz, Germany; sicqu@uni-mainz.de (S.Q.);; 2 Department of Urology and Pediatric Urology, University Medical Center, Johannes Gutenberg University, 55131 Mainz, Germany; zhuyewen.de@gmail.com

**Keywords:** serum iron status, age-related macular degeneration, retinitis pigmentosa, diabetic retinopathy, Mendelian randomization

## Abstract

**Background**: Observational studies have noted that patients with certain retinal degenerative diseases exhibit iron disturbances in the retina or vitreous compared to healthy controls. However, the connection between serum iron status and these diseases remains unclear. This study aims to explore the potential causal relationship between serum iron status biomarkers and the development of age-related macular degeneration (AMD), retinitis pigmentosa (RP), and diabetic retinopathy (DR). **Methods**: A two-sample Mendelian randomization (MR) analysis was conducted to investigate the causal relationship between serum iron status and several retinal degenerative diseases. Genome-wide association study (GWAS) summary-level data were extracted from public GWAS databases. Inverse-variance weighting (IVW), MR-Egger regressions, Simple model, Weighted median, and Weight mode were used as MR methods. Sensitivity analysis was conducted to confirm the robustness of the results by examining horizontal pleiotropy and heterogeneity through MR-Egger intercept and leave-one-out analysis. **Results**: The MR analysis revealed causal relationships between genetically predicted serum iron status biomarkers and various retinal diseases. Transferrin was positively associated with the odds of AMD (whether dry or wet) (OR = 1.167, 95% CI = 1.045–1.304, *p* = 0.006) and wet AMD (OR = 1.194, 95% CI = 1.018–1.402, *p* = 0.030). Ferritin was negatively associated with the odds of wet AMD (OR = 0.555, 95% CI = 0.333–0.927, *p* = 0.024). Serum iron (OR = 0.508, 95% CI = 0.260–0.993, *p* = 0.048) and transferrin saturation (OR = 0.508, 95% CI = 0.260–0.993, *p* = 0.048) were negatively associated with the odds of RP. **Conclusions**: These findings provide evidence supporting a potential causal relationship between serum iron status and various retinal degenerative diseases, highlighting a direction for future research into the underlying mechanisms of these diseases.

## 1. Introduction

Retinal degenerative diseases are a complex and multifactorial group of disorders, which mainly include retinal vascular diseases, maculopathy, retinitis pigmentosa (RP), and retinal detachment. For this study, we focused on the most representative diseases among three major categories: age-related macular degeneration (AMD), RP, and diabetic retinopathy (DR). These diseases can lead to progressive vision loss and ultimately blindness, imposing a heavy burden on the quality of life of patients and economically on society. AMD is the leading cause of irreversible blindness of people over 60 in developed countries, with the number of global cases expected to reach 288 million by 2040 [1,2]. AMD can be divided into two forms: wet AMD, characterized by choroidal neovascularization (CNV), and dry AMD, characterized by geographic atrophy (GA) [3]. As a revolutionary method, anti-vascular endothelial growth factor (VEGF) therapy has alleviated the condition of patients with wet AMD to a great extent, but some patients do not respond adequately to the therapy [4,5]. Additionally, there is currently no effective treatment for dry AMD. RP is a group of inherited eye disorders that are dystrophic degenerative diseases of the photoreceptors and retinal pigment epithelium [6]. The patient develops the disease in childhood, with early symptoms of night blindness, followed by a gradual loss of peripheral vision fields and eventually complete blindness [7]. Although antioxidants, nutrients, and vasodilators are considered to delay the progression of RP, and gene therapy has become a new direction in RP treatment, there is currently no treatment that can reverse RP [8]. DR, the most common retinal vascular disease, is the leading cause of blindness among working-aged adults around the world [9]. Its global prevalence is projected to rise to 191 million by 2030 [10]. According to severity and development stage of the disease, it can be divided into non-proliferative DR (NPDR) and proliferative DR (PDR). Early intervention is key to preventing DR. For advanced disease, anti-VEGF therapy and panretinal photocoagulation can inhibit or prevent neovascularization, thereby slowing the progression of DR [11]. However, high costs and uncertain efficacy pose challenges. In conclusion, it is necessary to further explore the breakthroughs to provide new therapeutic strategies to treat these diseases.

Supplementation with multiple vitamins and nutrients, such as vitamins C, E, β-carotene, lutein/zeaxanthin, and zinc, have been shown to benefit certain retinal degenerative diseases, highlighting the important role of trace elements in alleviating these diseases [12]. Recently, iron and iron-related proteins have also attracted significant attention due to their involvement in these retinal degenerative diseases. Furthermore, as ferroptosis has been identified as an important pathological mechanism in some retinal degenerative diseases, alterations in serum iron status have become increasingly important, highlighting its role in disease progression beyond that of other trace elements. Iron is an essential trace mineral involved in many aspects of cellular activity, including oxygen transport, energy production, catalysis of metabolic events, and biosynthesis [13]. Recently, the role of iron and iron-related proteins in retinal degenerative diseases has attracted much attention. Studies have reported higher iron levels in the aqueous humor of AMD patients and the vitreous body of PDR patients compared to controls [14,15,16]. Furthermore, iron overload and elevated iron metabolism-related proteins were found in the retinas of RP animal models (retinal degeneration 10 mice) [17]. However, not all studies have shown consistent results. In a case–control study, there was no significant difference in plasma iron levels between AMD patients and controls [18]. In another case–control study, the medians of serum iron and ferritin were found lower in DR patients than controls, but without statistical significance [18]. These contradictory results suggest that observational studies alone are insufficient to establish a link between serum iron status and retinal degenerative diseases. To provide clear targets for future prevention and treatment of these diseases, we used Mendelian randomization analysis to elucidate their causal relationships.

The body’s iron storage, transport, and overall iron metabolism are typically assessed through serum iron status [19]. The primary indicators used in clinical practice include serum iron, ferritin, transferrin saturation, and serum levels of transferrin. Serum iron reflects the level of free iron in circulation but can fluctuate physiologically [20]. Ferritin, an iron storage protein, indicates the body’s iron reserves and is the most sensitive marker for iron deficiency or overload [21]. Transferrin saturation, calculated as the ratio of serum iron to total iron-binding capacity, provides insight into iron transport efficiency [22]. Transferrin is a glycoprotein responsible for delivering ferric ions. Its serum level reflects iron transport and utilization, making it a crucial biomarker for assessing iron metabolism [23]. Together, these biomarkers offer a comprehensive evaluation of iron homeostasis, critical for diagnosing iron-related disorders.

Mendelian randomization (MR) is a novel epidemiological approach used to predict causal associations between exposure and disease outcomes. MR analysis utilizes genetic variants as instrumental variables (IVs) and derives information from genome-wide association studies (GWAS), making inferences based on single-nucleotide polymorphisms (SNPs) [24]. Because genetic variation satisfies the rules of random segregation of alleles from parents to their offspring, MR is less susceptible to population confounding factors than traditional observational studies [25]. In recent years, an increasing number of MR studies have focused on retinal degenerative diseases and revealed causative risk factors for the diseases. In this study, we conducted a two-sample MR analysis to evaluate the potential causality of four serum iron status biomarkers (serum iron, ferritin, transferrin saturation, and transferrin) with the risk of AMD, RP, and DR.

## 2. Materials and Methods

### 2.1. Study Design

**Figure 1:**
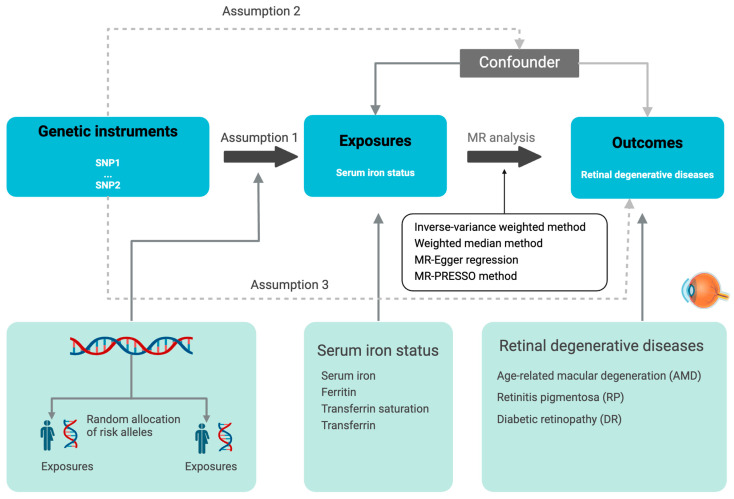
The principles of the MR analysis. SNP, single-nucleotide polymorphism; MR, Mendelian randomization. The figure was created using BioRender.com (https://biorender.com/, accessed on 3 September 2024).

We investigated the causal effects of serum iron status on AMD, RP, and DR by MR analysis using GWAS summary data. As shown in Figure 1, this MR study was based on three basic assumptions, including (1) relevance assumption: the IVs must be strongly associated with the exposure (serum iron status); (2) independence assumption: the IVs should not be correlated with other potential confounders; (3) restriction assumption: the IVs should affect the outcome solely through the exposure, and have no direct association with the outcome (AMD, RP, and DR) [26]. Based on the assumptions, our study aimed to provide evidence for the potential causal relationship between serum iron status and the development of the mentioned retinal degenerative diseases.

### 2.2. Data Sources for Serum Iron Status Biomarkers

Summary-level data for four serum iron status biomarkers including serum iron (ieu-a-1049), ferritin (ieu-a-1050), transferrin saturation (ieu-a-1051), and transferrin (ieu-a-1052) were obtained from the online platform of the Integrative Epidemiology Unit (IEU) open GWAS project (https://gwas.mrcieu.ac.uk/, accessed on 7 April 2024). Genetic data on serum iron status were sourced from 23,986 European individuals gathered from 11 cohorts of 9 participating centers [27]. The SNPs of the serum iron status biomarker data were visualized using the R package “qqman” (version 0.1.9).

### 2.3. Data Sources for AMD, RP, and DR

Summary-level data for AMD (including AMD (whether dry or wet), wet AMD, and dry AMD), RP, and DR (including DR, PDR, and NPDR) were obtained from the online platform of the IEU open GWAS project (https://gwas.mrcieu.ac.uk/). The AMD (wet and dry) GWAS dataset (ID: finn-b-H7_AMD) comprised 209,122 individuals, including 3763 cases and 205,359 controls, for a total of 16,380,424 SNPs. The wet AMD GWAS dataset (ID: finn-b-WET_AMD) comprised 208,715 individuals, including 2114 cases and 206,601 controls, for a total of 16,380,422 SNPs. The dry AMD GWAS dataset (ID: finn-b-DRY_AMD) comprised 208,690 individuals, including 2469 cases and 206,221 controls, for a total of 16,380,423 SNPs. Similarly, the RP GWAS dataset (ID: ebi-a-GCST90018912) comprised 126,454 individuals, including 121 cases and 126,333 controls, for a total of 22,242,822 SNPs. Finally, the DR GWAS dataset (ID: finn-b-DM_RETINOPATHY) involved 150,642 individuals, including 14,584 cases and 202,082 controls, for a total of 16,380,459 SNPs. The PDR GWAS dataset (ID: finn-b-DM_RETINA_PROLIF) involved 212,889 individuals, including 8681 cases and 204,208 controls, for a total of 16,380,460 SNPs. The NPDR GWAS dataset (ID: finn-b-DM_BCKGRND_RETINA_NONPROLIF) involved 204,663 individuals, including 455 cases and 204,208 controls, for a total of 16,380,42 SNPs. All participants in these datasets were of European ancestry.

### 2.4. Selection of IVs

First, SNPs strongly associated with the exposures at the genome-wide significance threshold (*p* < 5 × 10^−8^) were selected. However, because of the limited number of SNPs meeting this threshold for most serum iron status biomarkers, SNPs associated with exposure at a slightly lower threshold (*p* < 5 × 10^−6^) were chosen. This approach increased the number of IVs and strengthened the statistical power of the MR analysis while maintaining validity. To ensure the independence of the selected IVs, we implemented a rigorous clumping procedure to exclude SNPs with strong linkage disequilibrium, with r^2^ < 0.001 and a window size of 10,000 kb. This approach minimizes bias from potential SNP interactions and ensures that each IV independently represents a specific genetic variant. Additionally, the F statistic was calculated for each SNP to assess the robustness and strength of the selected instruments. SNPs with an F statistic > 10 were considered powerful IVs and included in further MR analysis. Furthermore, R package ieugwasr (V 0.1.5) was used to filter out SNPs associated with the outcome and potential confounding factors. All well-established risk factors for AMD, RP, and DR, such as smoking, hypertension, diabetes mellitus, and cardiovascular disease, were considered potential confounders and excluded from the analysis [28,29]. Finally, exposure and outcome data were harmonized, inferring positive strand alleles using allele frequencies for palindromic SNPs to ensure consistent matching of effect alleles.

### 2.5. Mendelian Randomization Analysis and Sensitivity Analysis

In this study, MR analyses were conducted using the “Two-Sample MR” R package (V.0.5.7) in R (V.4.3.0). The odds ratio (OR) coupled with its associated 95% confidence interval (CI) were the primary metrics for effect assessment. The inverse variance weighted (IVW) method was used as the primary analysis to estimate causal associations between serum iron status biomarkers and AMD, RP, and DR. The IVW method, which uses the inverse variances of IVs as weights, assumes that all IVs are valid, and provides accurate results in the absence of horizontal pleiotropy and heterogeneity [30,31]. Heterogeneity among genetic instruments was evaluated using Cochran’s Q statistic, with *p* > 0.05 indicating no significant heterogeneity [32]. For sensitivity exploration, MR-Egger intercept and leave-one-out analysis were adopted to the IVW. The MR-Egger regression was used to identify potential pleiotropy and evaluate the impact of pleiotropy on the risk estimation of the intercept test, and *p* > 0.05 indicates no pleiotropy [33]. Leave-one-out analysis was used to identify SNPs with potential impacts and evaluate the reliability of the results [34].

## 3. Results

### 3.1. Overview of Genetic IVs for Exposure

**Figure 2:**
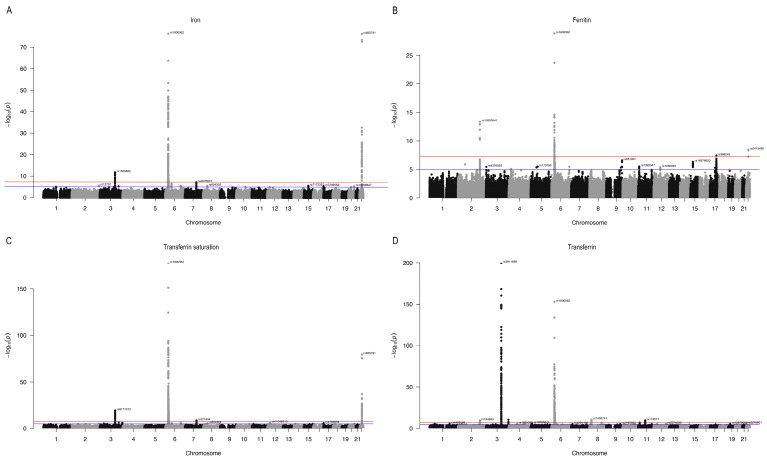
Manhattan plot showing SNPs associated with serum iron (**A**), ferritin (**B**), transferrin saturation (**C**) and transferrin (**D**). The *X*-axis represents chromosomes from 1 to 22, and the *Y*-axis represents the results of the association analysis, represented by −log10 (*p*). Each point represents its position on the chromosome, and the higher the point, the more significant the association of the gene or SNP. The blue line parallel to the *X*-axis refers to the “suggestive significance threshold”, and corresponds to a *p*-value threshold of 5 × 10^−5^. The red line parallel to the *X*-axis refers to the “genome-wide significance threshold”, and corresponds to a *p*-value threshold of 5 × 10^−8^.

First, we visualized the SNPs of the exposed data (including serum iron, ferritin, transferrin saturation, and transferrin) in the Manhattan plot (Figure 2). In the MR analysis, rigorous selection generated 47 SNPs as IVs for total AMD (both dry and wet forms), 43 SNPs for wet AMD, and 47 SNPs for dry AMD. Additionally, 51 SNPs were selected as IVs for RP. Furthermore, there were 41 SNPs selected as IVs for DR, 30 SNPs selected for PDR, and 35 SNPs selected for NPDR. Details of the SNPs identified for different serum iron status biomarkers are presented in Table S1. SNPs that did not pass the Mendelian hypothesis test were excluded from further analysis and are listed in Table S2. The combined instrument F statistic values for all SNPs exceeded the threshold of 10 (minimum = 21.02, maximum = 1269.06), effectively avoiding weak instrument bias and strongly predicting these retinal degenerative diseases in the MR analysis [35].

### 3.2. The Genetic Instruments and Risk Factors of AMD, RP, and DR

To explore potential risk factors affecting the interaction between serum iron status and AMD, RP, and DR, we examined whether high serum iron statuses were related to any underlying confounding factors. SNPs associated with outcomes were excluded from further analysis using the R package ieugwasr (V 0.1.5), and all removed SNPs were documented in Table S2. As a result, the number of IVs ranged from 5 to 19 for serum iron status.

### 3.3. MR Estimates

**Figure 3:**
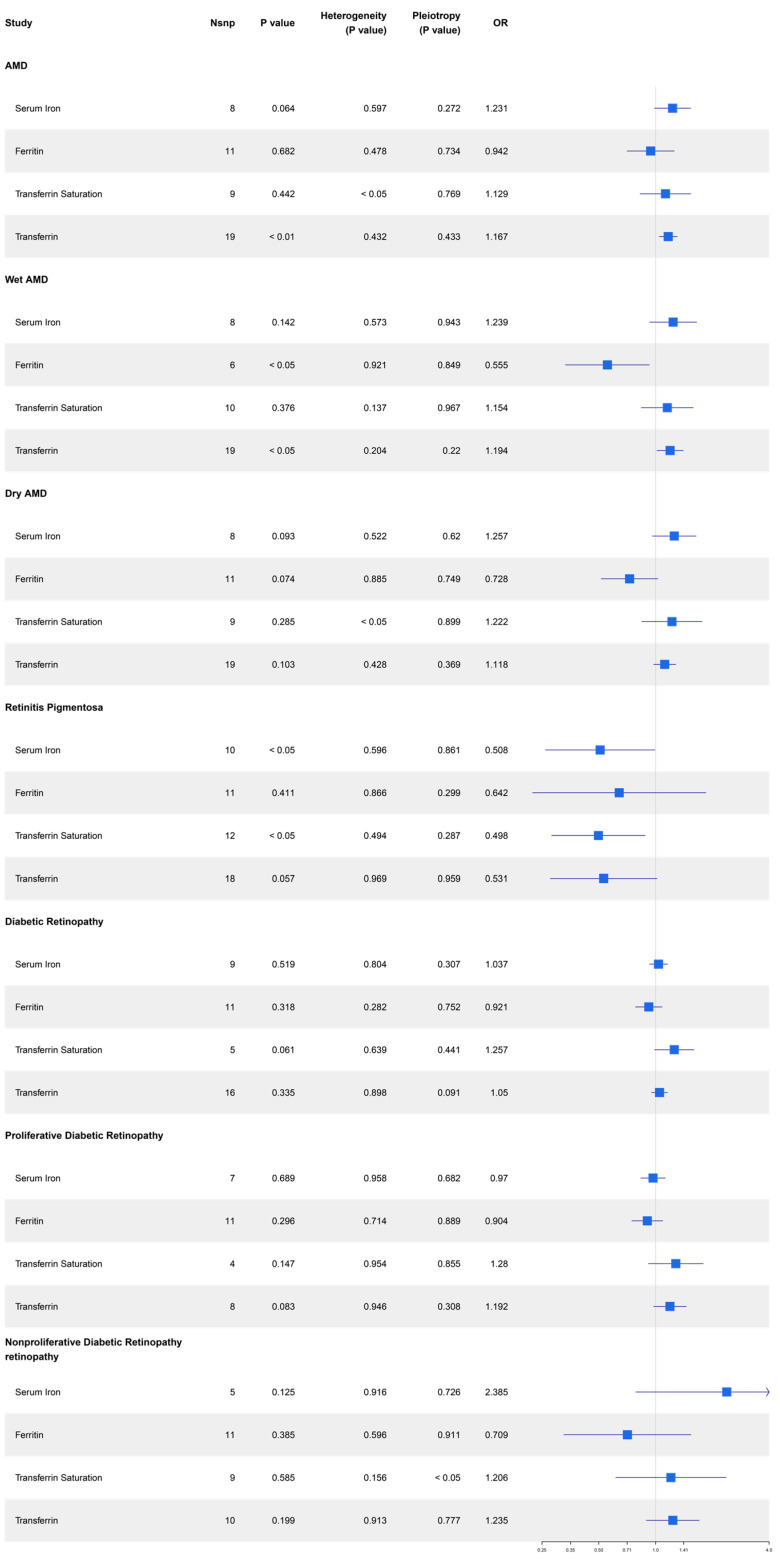
Forest plots estimated causal relationships between serum iron status indicators and three retinol degenerative diseases. Nsnp, number of single-nucleotide polymorphisms; OR, odds ratios.

**Figure 4:**
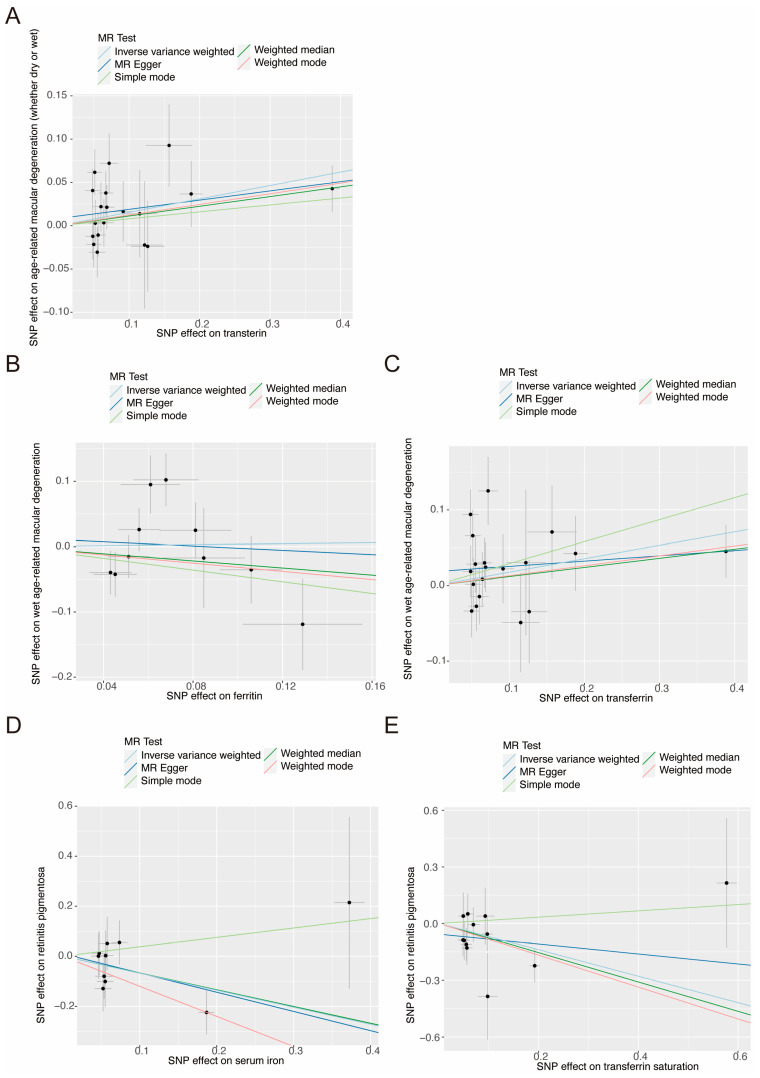
The scatter plots illustrating the associations between transferrin and AMD (whether dry or wet) (**A**), between ferritin and wet AMD (**B**), between transferrin and wet AMD (**C**), between serum iron and RP (**D**), and between transferrin saturation and RP (**E**). The five methods used in this study are depicted including IVW, MR-Egger, Simple model, Weighted median, and Weight mode. The vertical axis in scatter plots represents the effect of selected SNPs on the outcome, the horizontal axis is the effect of selected SNPs on the exposure, and each distinct point represents an instrumental SNP. The grey line indicates the simple mode approach, which calculates the mode of the SNP-specific causal estimates without weighting. SNP, single-nucleotide polymorphisms; MR, Mendelian randomization.

The MR analysis results for the effect of serum iron status on the risk of three retinal degenerative diseases are presented in Figure 3. The IVW analysis indicated a positive causal association between transferrin and AMD (whether dry or wet) (OR = 1.167, 95% CI = 1.045–1.304, *p* = 0.006), and between transferrin and wet AMD (OR = 1.194, 95% CI = 1.018–1.402, *p* = 0.030). Conversely, there was a negative causal association between ferritin and wet AMD (OR = 0.555, 95% CI = 0.333–0.927, *p* = 0.024). Furthermore, the IVW analysis indicated a negative causal association between serum iron and RP (OR = 0.508, 95% CI = 0.260–0.993, *p* = 0.048), and between transferrin saturation and RP (OR = 0.508, 95% CI = 0.260–0.993, *p* = 0.048). However, no causal association was identified between serum iron status biomarkers and the risk of dry AMD or any types of DR. Scatter plots provided a more intuitive visualization for the results (*p*  <  0.05) in Figure 4 and Figure S1.

### 3.4. Sensitivity Analyses

**Figure 5:**
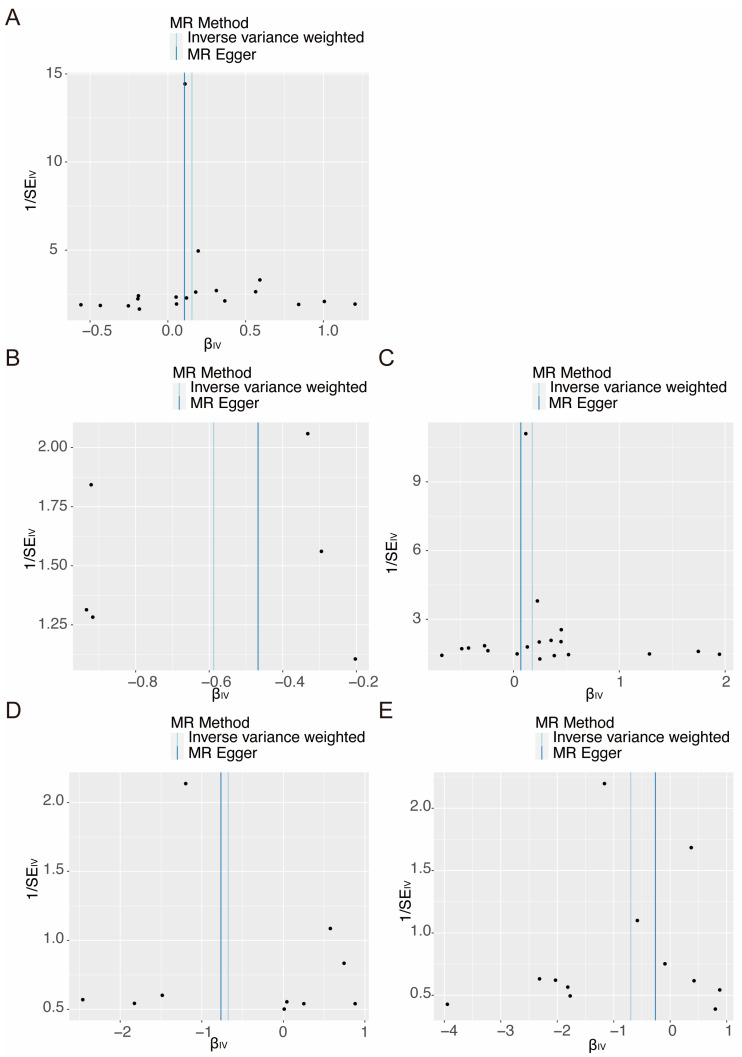
The funnel plots employed to evaluate the causal association between serum iron status and selected retinal degenerative diseases in MR analysis. (**A**) Transferrin on AMD (whether dry or wet), (**B**) ferritin on wet AMD, (**C**) transferrin on wet AMD, (**D**) serum iron on RP, and (**E**) transferrin saturation on RP. Black points represent individual SNPs. MR, Mendelian randomization; IV, instrumental variable; SE, standard error; SNP, single-nucleotide polymorphisms.

**Figure 6:**
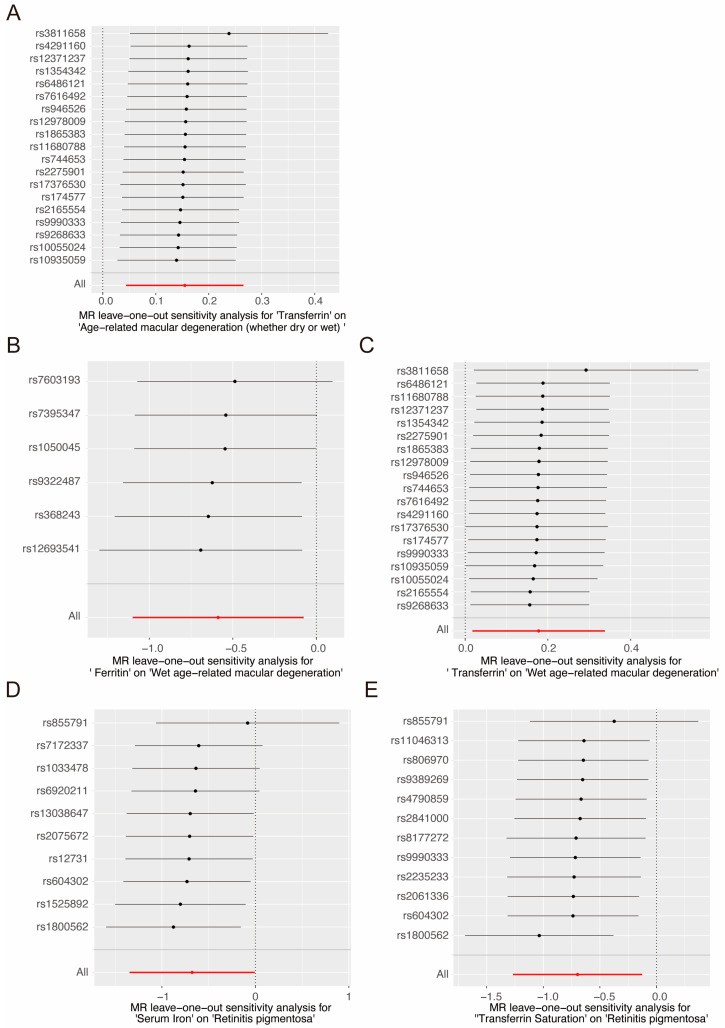
The leave-one-out analysis of the causal relationship between serum iron status and selected retinal degenerative diseases. (**A**) Transferrin on AMD (whether dry or wet), (**B**) ferritin on wet AMD, (**C**) transferrin on wet AMD, (**D**) serum iron on RP, and (**E**) transferrin saturation on RP. Black points represent the MR effect estimate when each specific SNP is omitted. Black lines are the confidence intervals for these estimates, showing variability when each SNP is excluded. Red line indicates the overall effect estimate with all SNPs included. Red point represents the overall effect estimate when all SNPs are included, showing the total causal effect without excluding any SNPs.

In the sensitivity analysis, no significant heterogeneity was detected in the selected instruments using Cochran’s Q test (*p* > 0.05; Table 1 and Table S3). The funnel plots also confirmed the absence of heterogeneity within the identified associations (Figure 5 and Figure S2). Additionally, no pleiotropy was detected in the P-Egger intercept of MR-Egger and the global test of MR-PRESSO (global test *p* > 0.05; Table 1 and Table S3). The leave-one-out analysis plot demonstrated that the results remained robust after sequentially excluding each SNP (Figure 6 and Figure S3). This confirmed that the results were driven by the overall pattern of associations rather than by any particular extreme SNP.

## 4. Discussion

Iron is one of the necessary minerals in the human body that is essential for various biochemical processes [36]. Its status can reflect overall health and is often associated with various diseases. For the first time, we applied MR to analyze the genetic causal relationship between four biomarkers of serum iron status and retinal degenerative diseases, including AMD, RP, and DM. Our findings suggested a positive causal association between transferrin and AMD, particularly wet AMD, while ferritin showed a negative causal association with wet AMD. Additionally, serum iron and transferrin saturation are negatively associated with RP. However, no causal relationship was identified between serum iron status biomarkers and the risk of dry AMD or any type of DR. These insights provided a novel understanding of the mechanisms underlying retinal degenerative diseases.

In our study, transferrin was found to be positively correlated with the occurrence of AMD (especially wet AMD). It is a protein expressed in many species, including humans, and primarily transports iron from the circulatory system to various tissues [37,38]. Transferrin mRNA and protein are more abundant in the retina than in other tissues [39]. In the retina, iron is transported into cells via endocytosis facilitated by transferrin receptors (TFR) located on the retinal cell surface [40]. Specifically, at the outer blood–retinal barrier (BRB), iron bound to transferrin in the choroidal capillaries is transported to the RPE via TFR1 on the basement membrane of the RPE. After entering the RPE cytoplasm, the iron is stored in melanosomes and ferritin [41]. According to Itay Chowers et al., transferrin mRNA and protein expression levels were elevated in the retinas of AMD patients compared to healthy controls [34]. Additionally, higher levels of soluble transferrin receptors in AMD patients further suggested abnormal transferrin changes during the disease [16]. Although transferrin does not normally cross the blood–retinal barrier (BRB), pathological conditions that disrupt the BRB allow for the entry of transferrin [42,43]. Furthermore, apo-transferrin, as an endogenous iron transporter, is allowed to cross the BRB [44]. Thus, serum transferrin levels may partially reflect intraocular levels, making it a potential predictor of disease progression.

Ferritin, another iron-related protein, was found to have a negative correlation with the occurrence of wet AMD. It is a cytosolic iron storage protein consisting of two subunits, ferritin heavy chain 1 (FTH1) and ferritin light chain (FTL) [37]. In a population-based case–control study of Koreans, multiple linear regression analysis showed that serum ferritin levels were closely associated with AMD [45]. However, these findings indicated higher serum ferritin levels in AMD patients than in controls, which is contrary to our conclusion. This discrepancy may arise from differences in ethnic groups and disease stages. Since cytoplasmic ferritin exerts a protective effect through its ability to chelate free iron, we propose that ferritin may help prevent the development of AMD. Although no causal relationship was found between ferritin and dry AMD in our MR analysis. A study showed that ferritin was present in the photoreceptor and internal limiting membrane regions of the macula with GA [46]. In another study, upregulation of FTH1 was also shown to ameliorate dry AMD-like pathology in a mouse model, suggesting that serum ferritin levels in healthy individuals may be a useful indicator for all types of AMD [47].

RP is the most common inherited retinal degenerative disease, characterized by the irreversible death of photoreceptors [48]. In addition to cell death caused by reduced oxygen consumption capacity, iron accumulation is also implicated in RP’s pathogenesis, with iron overload observed in animal models such as retinal degeneration in 10 mice and Royal College of Surgeons rats [17,49]. Our study suggested a negative causal relationship between serum iron levels and RP. This may be due to the local accumulation of iron, resulting in a decrease in circulating iron levels. Transferrin saturation was also found to be negatively causally associated with RP in our study. It is an indicator that is calculated as serum iron divided by total iron-binding capacity, and is useful in diagnosing abnormalities of iron metabolism [50]. Although the current research does not directly link transferrin saturation to RP, the positive impact of increased bound iron levels on RP reflected by this is consistent with our understanding of pathological mechanisms of this disease.

Regarding DR, no causal relationship was identified between serum iron status biomarkers and the risk of all types of DR. Contrary to our finding, a cohort study of NPDR patients indicated that patients with macular edema had significantly higher ferritin levels, and significantly lower serum iron and transferrin saturation levels than controls [51]. This discrepancy may be due to limitations in observational studies, such as confounding factors, retrospective design, and sample size.

Unlike other forms of cell death, ferroptosis is a novel iron-dependent regulated cell death thought to be involved in the pathogenesis of certain retinal degenerative diseases [52]. Excess iron deposition in the retina can be toxic, forming reactive oxygen species, inducing the Fenton reactions, and further promoting lipid peroxidation. When these oxidation cascade reactions exceed the antioxidant capacity of the cells, it can lead to cell damage or death [53]. In AMD, both subclinical BRB leakage in early AMD and severe BRB disruption in the late stage of exudative AMD can lead to potentially harmful plasma components from the circulation to enter the neurosensory retina of AMD patients and disrupt the intraocular serum iron status [38,54]. In RP, overexpression of a key ferroptosis factor (Gpx4) and application of ferroptosis inhibitors could work against retinal degeneration and promote cone photoreceptor survival [55]. Similarly, in DR, studies showed that ferroptosis-related biomarkers accumulated in both NPDR and PDR patients, and in addition, ferroptosis was found to be associated with microvascular dysfunction in PDR [56,57]. These findings demonstrated a driving role for ferroptosis in certain retinal degenerative diseases and suggested the potential value of measuring serum iron status biomarkers for early disease diagnosis.

However, our study has several limitations. First, the data we relied on are summary-level rather than individual-level data, and therefore, were unable to use potential factors such as age and gender to further stratify the relationship between serum iron status and mentioned retinal degenerative diseases. Additionally, different disease types or stages may respond differently to serum iron levels, as seen with BRB leakage in advanced AMD. Second, all study participants were of European ancestry, and serum iron status may vary across ethnic groups. For example, East Asians have higher concentrations of serum iron status indicators than other races [58]. Whether our findings can be generalized to all populations remains to be verified. Third, although we excluded most confounding factors that may be caused by high serum iron status, a few innate or environmental factors should not be ruled out due to the limited knowledge. Additionally, other serum iron status indicators not included in our study may be more relevant to AMD, RP, and DR, and require further exploration.

## 5. Conclusions

This study demonstrates a causal relationship between serum iron status and certain retinal degenerative diseases through Mendelian randomization analysis. The findings showed that changes in serum iron and transferrin saturation were causally linked to RP. Moreover, serum ferritin levels AMD, RP, and DR were causally associated with wet AMD, while serum transferrin levels were related to both forms of AMD, particularly wet AMD. These results underscore the importance of serum iron status for the early diagnosis of these diseases and suggest feasible methods that may simplify the prediction of related diseases. Furthermore, the study offers valuable insights for future studies into the underlying pathological mechanisms and potential treatment options.

## Figures and Tables

**Table 1:** The results of sensitivity tests for all initially significant associations (*p* < 0.05) between serum iron status indicators and selected retinal degenerative diseases. RSSobs, Observation of residual sum of squares.

Exposure	Outcome	Cochran’s Q Test				Egger Intercept Test		MR-PRESSO	
		Q-IVW	*p*-Value	MR Egger	*p*-Value	Egger- Intercept	*p*-Value	RSSobs	*p*-Value of Global Test
Transferrin	AMD (whether dry or wet)	18.363	0.432	17.692	0.409	0.008	0.433	21.945	0.494
Ferritin	Wet AMD	1.428	0.921	1.387	0.846	−0.009	0.849	2.193	0.918
Transferrin		13.613	0.137	13.610	0.093	0.001	0.967	15.380	0.230
Serum iron	Retinitis Pigmentosa	7.397	0.596	7.364	0.498	0.010	0.861	12.951	0.489
Transferrin Saturation		10.405	0.494	9.138	0.519	−0.057	0.287	15.065	0.437

## Data Availability

All datasets used in this study are available from the corresponding author upon reasonable request or can be downloaded at https://gwas.mrcieu.ac.uk/ (accessed on 6 April 2024).

## Supplementary Materials

The following supporting information can be downloaded at: https://www.mdpi.com/article/10.3390/nu16213747/s1, Table S1: Characteristics of SNPs used as instrumental variables for serum iron status in MR analysis; Table S2: The removed instrumental variables for serum iron status in MR analysis; Table S3: Cochran’s Q test, Egger intercept test and MR-PRESSO of serum iron status on retinal degenerative diseases in the MR analysis; Figure S1: Scatter plots of genetic correlations of serum iron status and retinal degenerative diseases using different MR methods; Figure S2: Leave-one-out analysis of the causal relationship between serum iron status and retinal degenerative diseases; Figure S3: Funnel plots of the causal relationship between serum iron status and retinal degenerative diseases.
