# Supplementary material for: Serum Iron Status and Retinal Degenerative Diseases: A Mendelian Randomization Study on AMD, RP, and DR
Source: Nutrients. 2024 Oct 31;16(21):3747. doi: 10.3390/nu16213747 (PMC11547415; doi:10.3390/nu16213747)
Supplement: Supplementary file 1 [file nutrients-16-03747-s001.zip › nutrients-3229228-supplementary.pdf]

Disease	SNP	effect_ allele	other_ allele	β	SE	p-value	F-statistics
**AMD (whether dry or wet)**	**Iron**						
	rs1033478	C	T	-0.055	0.011	3.63E-07	26.029
	rs12731	A	G	0.047	0.010	2.52E-06	22.373
	rs13038647	C	T	0.046	0.010	3.80E-06	21.437
	rs1525892	A	G	0.074	0.010	1.65E-12	50.083
	rs2075672	G	A	0.056	0.010	5.95E-08	29.244
	rs604302	C	T	-0.058	0.012	3.07E-06	21.653
	rs7172337	C	T	0.052	0.011	2.63E-06	22.200
	rs855791	G	A	0.187	0.010	4.31E-77	342.067
	**Ferritin**						
	rs1050045	C	T	-0.043	0.009	4.12E-06	21.023
	rs12693541	T	C	-0.106	0.014	4.18E-14	57.327
	rs16976620	A	G	-0.081	0.016	4.52E-07	25.374
	rs173780	A	G	-0.068	0.015	3.35E-06	21.629
	rs1752162	T	C	0.061	0.013	4.80E-06	21.036
	rs2413450	C	T	0.056	0.010	3.57E-09	34.624
	rs368243	C	T	-0.051	0.009	3.80E-08	30.309
	rs4376025	T	C	0.046	0.010	3.62E-06	21.437
	rs7395347	T	C	0.045	0.010	3.20E-06	21.714
	rs7603193	T	C	-0.129	0.027	1.38E-06	23.343
	rs9322487	A	G	0.085	0.018	3.47E-06	21.473
	**Transferrin Saturation**						
	rs11046313	A	C	-0.056	0.011	6.86E-07	24.647
	rs2061336	G	A	0.093	0.018	1.99E-07	26.936
	rs2235233	C	T	-0.069	0.013	8.81E-08	28.610
	rs2841000	T	C	-0.051	0.011	2.78E-06	22.038
	rs4790859	G	A	-0.048	0.010	2.29E-06	22.423
	rs604302	C	T	-0.058	0.012	3.26E-06	21.803
	rs8177272	A	G	-0.097	0.011	5.52E-20	83.740
	rs855791	G	A	0.192	0.010	3.50E-80	361.753
	rs9990333	T	C	0.049	0.010	7.37E-07	24.298
	**Transferrin**						
	rs10055024	T	C	0.051	0.010	8.98E-07	24.331
	rs10935059	T	G	-0.157	0.033	2.02E-06	22.577
	rs11680788	T	C	-0.115	0.025	4.57E-06	21.028
	rs12371237	A	C	-0.050	0.011	3.48E-06	21.662
	rs12978009	A	G	-0.064	0.014	3.17E-06	21.643
	rs1354342	G	A	-0.126	0.024	1.28E-07	27.970
	rs17376530	T	C	-0.188	0.017	5.43E-30	129.960
	rs174577	A	C	0.068	0.011	1.90E-10	40.864
	rs1865383	T	G	-0.052	0.011	2.17E-06	22.347
	rs2165554	T	C	-0.048	0.011	4.19E-06	21.160
	rs2275901	A	G	0.060	0.013	1.77E-06	22.751
	rs3811658	T	C	0.388	0.011	1.00E-200	1269.059
	rs4291160	G	T	0.055	0.012	3.68E-06	21.567
	rs6486121	T	C	-0.056	0.011	1.04E-07	28.546
	rs744653	T	C	0.092	0.014	2.00E-10	40.464
	rs7616492	A	G	-0.049	0.011	4.02E-06	21.336
	rs9268633	G	A	0.072	0.013	2.31E-08	31.378
	rs946526	C	T	0.122	0.026	2.98E-06	21.849
	rs9990333	T	C	-0.067	0.010	3.01E-11	44.005
**Wet AMD**	**Iron**						
	rs1033478	C	T	-0.055	0.011	3.63E-07	26.029

	rs12731	A	G	0.047	0.010	2.52E-06	22.373
	rs13038647	C	T	0.046	0.010	3.80E-06	21.437
	rs1525892	A	G	0.074	0.010	1.65E-12	50.083
	rs2075672	G	A	0.056	0.010	5.95E-08	29.244
	rs604302	C	T	-0.058	0.012	3.07E-06	21.653
	rs7172337	C	T	0.052	0.011	2.63E-06	22.200
	rs855791	G	A	0.187	0.010	4.31E-77	342.067
	**Ferritin**						
	rs1050045	C	T	-0.043	0.009	4.12E-06	21.023
	rs12693541	T	C	-0.106	0.014	4.18E-14	57.327
	rs368243	C	T	-0.051	0.009	3.80E-08	30.309
	rs7395347	T	C	0.045	0.010	3.20E-06	21.714
	rs7603193	T	C	-0.129	0.027	1.38E-06	23.343
	rs9322487	A	G	0.085	0.018	3.47E-06	21.473
	**Transferrin Saturation**						
	rs11046313	A	C	-0.056	0.011	6.86E-07	24.647
	rs2061336	G	A	0.093	0.018	1.99E-07	26.936
	rs2235233	C	T	-0.069	0.013	8.81E-08	28.610
	rs2841000	T	C	-0.051	0.011	2.78E-06	22.038
	rs4790859	G	A	-0.048	0.010	2.29E-06	22.423
	rs604302	C	T	-0.058	0.012	3.26E-06	21.803
	rs806970	T	C	-0.098	0.020	6.93E-07	24.596
	rs8177272	A	G	-0.097	0.011	5.52E-20	83.740
	rs855791	G	A	0.192	0.010	3.50E-80	361.753
	rs9990333	T	C	0.049	0.010	7.37E-07	24.298
	**Transferrin**						
	rs10055024	T	C	0.051	0.010	8.98E-07	24.331
	rs10935059	T	G	-0.157	0.033	2.02E-06	22.577
	rs11680788	T	C	-0.115	0.025	4.57E-06	21.028
	rs12371237	A	C	-0.050	0.011	3.48E-06	21.662
	rs12978009	A	G	-0.064	0.014	3.17E-06	21.643
	rs1354342	G	A	-0.126	0.024	1.28E-07	27.970
	rs17376530	T	C	-0.188	0.017	5.43E-30	129.960
	rs174577	A	C	0.068	0.011	1.90E-10	40.864
	rs1865383	T	G	-0.052	0.011	2.17E-06	22.347
	rs2165554	T	C	-0.048	0.011	4.19E-06	21.160
	rs2275901	A	G	0.060	0.013	1.77E-06	22.751
	rs3811658	T	C	0.388	0.011	1.00E-200	1269.059
	rs4291160	G	T	0.055	0.012	3.68E-06	21.567
	rs6486121	T	C	-0.056	0.011	1.04E-07	28.546
	rs744653	T	C	0.092	0.014	2.00E-10	40.464
	rs7616492	A	G	-0.049	0.011	4.02E-06	21.336
	rs9268633	G	A	0.072	0.013	2.31E-08	31.378
	rs946526	C	T	0.122	0.026	2.98E-06	21.849
	rs9990333	T	C	-0.067	0.010	3.01E-11	44.005
**Dry AMD**	**Iron**						
	rs1033478	C	T	-0.055	0.011	3.63E-07	26.029
	rs12731	A	G	0.047	0.010	2.52E-06	22.373
	rs13038647	C	T	0.046	0.010	3.80E-06	21.437
	rs1525892	A	G	0.074	0.010	1.65E-12	50.083

	rs2075672	G	A	0.056	0.010	5.95E-08	29.244
	rs604302	C	T	-0.058	0.012	3.07E-06	21.653
	rs7172337	C	T	0.052	0.011	2.63E-06	22.200
	rs855791	G	A	0.187	0.010	4.31E-77	342.067
	**Ferritin**						
	rs1050045	C	T	-0.043	0.009	4.12E-06	21.023
	rs12693541	T	C	-0.106	0.014	4.18E-14	57.327
	rs16976620	A	G	-0.081	0.016	4.52E-07	25.374
	rs173780	A	G	-0.068	0.015	3.35E-06	21.629
	rs1752162	T	C	0.061	0.013	4.80E-06	21.036
	rs2413450	C	T	0.056	0.010	3.57E-09	34.624
	rs368243	C	T	-0.051	0.009	3.80E-08	30.309
	rs4376025	T	C	0.046	0.010	3.62E-06	21.437
	rs7395347	T	C	0.045	0.010	3.20E-06	21.714
	rs7603193	T	C	-0.129	0.027	1.38E-06	23.343
	rs9322487	A	G	0.085	0.018	3.47E-06	21.473
	**Transferrin Saturation**						
	rs11046313	A	C	-0.056	0.011	6.86E-07	24.647
	rs2061336	G	A	0.093	0.018	1.99E-07	26.936
	rs2235233	C	T	-0.069	0.013	8.81E-08	28.610
	rs2841000	T	C	-0.051	0.011	2.78E-06	22.038
	rs4790859	G	A	-0.048	0.010	2.29E-06	22.423
	rs604302	C	T	-0.058	0.012	3.26E-06	21.803
	rs8177272	A	G	-0.097	0.011	5.52E-20	83.740
	rs855791	G	A	0.192	0.010	3.50E-80	361.753
	rs9990333	T	C	0.049	0.010	7.37E-07	24.298
	**Transferrin**						
	rs10055024	T	C	0.051	0.010	8.98E-07	24.331
	rs10935059	T	G	-0.157	0.033	2.02E-06	22.577
	rs11680788	T	C	-0.115	0.025	4.57E-06	21.028
	rs12371237	A	C	-0.050	0.011	3.48E-06	21.662
	rs12978009	A	G	-0.064	0.014	3.17E-06	21.643
	rs1354342	G	A	-0.126	0.024	1.28E-07	27.970
	rs17376530	T	C	-0.188	0.017	5.43E-30	129.960
	rs174577	A	C	0.068	0.011	1.90E-10	40.864
	rs1865383	T	G	-0.052	0.011	2.17E-06	22.347
	rs2165554	T	C	-0.048	0.011	4.19E-06	21.160
	rs2275901	A	G	0.060	0.013	1.77E-06	22.751
	rs3811658	T	C	0.388	0.011	1.00E-200	1269.059
	rs4291160	G	T	0.055	0.012	3.68E-06	21.567
	rs6486121	T	C	-0.056	0.011	1.04E-07	28.546
	rs744653	T	C	0.092	0.014	2.00E-10	40.464
	rs7616492	A	G	-0.049	0.011	4.02E-06	21.336
	rs9268633	G	A	0.072	0.013	2.31E-08	31.378
	rs946526	C	T	0.122	0.026	2.98E-06	21.849
	rs9990333	T	C	-0.067	0.010	3.01E-11	44.005
**Retinitis Pigmentosa**	**Iron**						
	rs1033478	C	T	-0.055	0.011	3.63E-07	26.029
	rs12731	A	G	0.047	0.010	2.52E-06	22.373
	rs13038647	C	T	0.046	0.010	3.80E-06	21.437

	rs1525892	A	G	0.074	0.010	1.65E-12	50.083
	rs1800562	A	G	0.372	0.020	3.96E-77	346.704
	rs2075672	G	A	0.056	0.010	5.95E-08	29.244
	rs604302	C	T	-0.058	0.012	3.07E-06	21.653
	rs6920211	C	T	0.054	0.012	3.14E-06	21.805
	rs7172337	C	T	0.052	0.011	2.63E-06	22.200
	rs855791	G	A	0.187	0.010	4.31E-77	342.067
	**Ferritin**						
	rs1050045	C	T	-0.043	0.009	4.12E-06	21.023
	rs16976620	A	G	-0.081	0.016	4.52E-07	25.374
	rs173780	A	G	-0.068	0.015	3.35E-06	21.629
	rs1752162	T	C	0.061	0.013	4.80E-06	21.036
	rs1800562	A	G	0.211	0.019	1.42E-29	127.316
	rs2413450	C	T	0.056	0.010	3.57E-09	34.624
	rs368243	C	T	-0.051	0.009	3.80E-08	30.309
	rs4376025	T	C	0.046	0.010	3.62E-06	21.437
	rs651007	T	C	-0.060	0.012	2.54E-07	26.487
	rs7395347	T	C	0.045	0.010	3.20E-06	21.714
	rs7603193	T	C	-0.129	0.027	1.38E-06	23.343
	**Transferrin Saturation**						
	rs11046313	A	C	-0.056	0.011	6.86E-07	24.647
	rs1800562	A	G	0.577	0.020	1.52E-178	808.464
	rs2061336	G	A	0.093	0.018	1.99E-07	26.936
	rs2235233	C	T	-0.069	0.013	8.81E-08	28.610
	rs2841000	T	C	-0.051	0.011	2.78E-06	22.038
	rs4790859	G	A	-0.048	0.010	2.29E-06	22.423
	rs604302	C	T	-0.058	0.012	3.26E-06	21.803
	rs806970	T	C	-0.098	0.020	6.93E-07	24.596
	rs8177272	A	G	-0.097	0.011	5.52E-20	83.740
	rs855791	G	A	0.192	0.010	3.50E-80	361.753
	rs9389269	C	T	0.055	0.011	9.78E-07	24.028
	rs9990333	T	C	0.049	0.010	7.37E-07	24.298
	**Transferrin**						
	rs10055024	T	C	0.051	0.010	8.98E-07	24.331
	rs10935059	T	G	-0.157	0.033	2.02E-06	22.577
	rs1110446	T	C	0.056	0.012	4.18E-06	21.296
	rs11680788	T	C	-0.115	0.025	4.57E-06	21.028
	rs12371237	A	C	-0.050	0.011	3.48E-06	21.662
	rs12978009	A	G	-0.064	0.014	3.17E-06	21.643
	rs1354342	G	A	-0.126	0.024	1.28E-07	27.970
	rs17376530	T	C	-0.188	0.017	5.43E-30	129.960
	rs1800562	A	G	-0.550	0.021	1.26E-153	698.179
	rs1865383	T	G	-0.052	0.011	2.17E-06	22.347
	rs2165554	T	C	-0.048	0.011	4.19E-06	21.160
	rs2275901	A	G	0.060	0.013	1.77E-06	22.751
	rs4291160	G	T	0.055	0.012	3.68E-06	21.567
	rs6486121	T	C	-0.056	0.011	1.04E-07	28.546
	rs744653	T	C	0.092	0.014	2.00E-10	40.464
	rs7616492	A	G	-0.049	0.011	4.02E-06	21.336
	rs946526	C	T	0.122	0.026	2.98E-06	21.849

	rs9990333	T	C	-0.067	0.010	3.01E-11	44.005
**Diabetic Retinopathy**	**Iron**						
	rs1033478	C	T	-0.055	0.011	3.63E-07	26.029
	rs12731	A	G	0.047	0.010	2.52E-06	22.373
	rs13038647	C	T	0.046	0.010	3.80E-06	21.437
	rs1525892	A	G	0.074	0.010	1.65E-12	50.083
	rs2075672	G	A	0.056	0.010	5.95E-08	29.244
	rs604302	C	T	-0.058	0.012	3.07E-06	21.653
	rs6920211	C	T	0.054	0.012	3.14E-06	21.805
	rs7172337	C	T	0.052	0.011	2.63E-06	22.200
	**Ferritin**						
	rs1050045	C	T	-0.043	0.009	4.12E-06	21.023
	rs12693541	T	C	-0.106	0.014	4.18E-14	57.327
	rs16976620	A	G	-0.081	0.016	4.52E-07	25.374
	rs173780	A	G	-0.068	0.015	3.35E-06	21.629
	rs1752162	T	C	0.061	0.013	4.80E-06	21.036
	rs2413450	C	T	0.056	0.010	3.57E-09	34.624
	rs368243	C	T	-0.051	0.009	3.80E-08	30.309
	rs4376025	T	C	0.046	0.010	3.62E-06	21.437
	rs7395347	T	C	0.045	0.010	3.20E-06	21.714
	rs7603193	T	C	-0.129	0.027	1.38E-06	23.343
	rs9322487	A	G	0.085	0.018	3.47E-06	21.473
	**Transferrin Saturation**						
	rs11046313	A	C	-0.056	0.011	6.86E-07	24.647
	rs2061336	G	A	0.093	0.018	1.99E-07	26.936
	rs2841000	T	C	-0.051	0.011	2.78E-06	22.038
	rs604302	C	T	-0.058	0.012	3.26E-06	21.803
	rs9389269	C	T	0.055	0.011	9.78E-07	24.028
	**Transferrin**						
	rs10055024	T	C	0.051	0.010	8.98E-07	24.331
	rs10935059	T	G	-0.157	0.033	2.02E-06	22.577
	rs11680788	T	C	-0.115	0.025	4.57E-06	21.028
	rs12371237	A	C	-0.050	0.011	3.48E-06	21.662
	rs12978009	A	G	-0.064	0.014	3.17E-06	21.643
	rs1354342	G	A	-0.126	0.024	1.28E-07	27.970
	rs1495741	A	G	0.083	0.012	1.57E-11	45.729
	rs17376530	T	C	-0.188	0.017	5.43E-30	129.960
	rs1865383	T	G	-0.052	0.011	2.17E-06	22.347
	rs2165554	T	C	-0.048	0.011	4.19E-06	21.160
	rs2275901	A	G	0.060	0.013	1.77E-06	22.751
	rs4291160	G	T	0.055	0.012	3.68E-06	21.567
	rs6486121	T	C	-0.056	0.011	1.04E-07	28.546
	rs744653	T	C	0.092	0.014	2.00E-10	40.464
	rs7616492	A	G	-0.049	0.011	4.02E-06	21.336
	rs9990333	T	C	-0.067	0.010	3.01E-11	44.005
**Proliferative Diabetic Retinopathy**	**Iron**						
	rs12731	A	G	0.047	0.010	2.52E-06	22.373
	rs13038647	C	T	0.046	0.010	3.80E-06	21.437
	rs1525892	A	G	0.074	0.010	1.65E-12	50.083
	rs604302	C	T	-0.058	0.012	3.07E-06	21.653

	rs6920211	C	T	0.054	0.012	3.14E-06	21.805
	rs7172337	C	T	0.052	0.011	2.63E-06	22.200
	rs855791	G	A	0.187	0.010	4.31E-77	342.067
	**Ferritin**						
	rs1050045	C	T	-0.043	0.009	4.12E-06	21.023
	rs12693541	T	C	-0.106	0.014	4.18E-14	57.327
	rs16976620	A	G	-0.081	0.016	4.52E-07	25.374
	rs173780	A	G	-0.068	0.015	3.35E-06	21.629
	rs1752162	T	C	0.061	0.013	4.80E-06	21.036
	rs2413450	C	T	0.056	0.010	3.57E-09	34.624
	rs368243	C	T	-0.051	0.009	3.80E-08	30.309
	rs4376025	T	C	0.046	0.010	3.62E-06	21.437
	rs7395347	T	C	0.045	0.010	3.20E-06	21.714
	rs7603193	T	C	-0.129	0.027	1.38E-06	23.343
	rs9322487	A	G	0.085	0.018	3.47E-06	21.473
	**Transferrin Saturation**						
	rs11046313	A	C	-0.056	0.011	6.86E-07	24.647
	rs2061336	G	A	0.093	0.018	1.99E-07	26.936
	rs2841000	T	C	-0.051	0.011	2.78E-06	22.038
	rs9990333	T	C	0.049	0.010	7.37E-07	24.298
	**Transferrin**						
	rs10935059	T	G	-0.157	0.033	2.02E-06	22.577
	rs11680788	T	C	-0.115	0.025	4.57E-06	21.028
	rs12371237	A	C	-0.050	0.011	3.48E-06	21.662
	rs12978009	A	G	-0.064	0.014	3.17E-06	21.643
	rs1865383	T	G	-0.052	0.011	2.17E-06	22.347
	rs2275901	A	G	0.060	0.013	1.77E-06	22.751
	rs6486121	T	C	-0.056	0.011	1.04E-07	28.546
	rs7616492	A	G	-0.049	0.011	4.02E-06	21.336
**Nonproliferative Diabetic Retinopathy**	**Iron**						
	rs1033478	C	T	-0.055	0.011	3.63E-07	26.029
	rs1525892	A	G	0.074	0.010	1.65E-12	50.083
	rs2075672	G	A	0.056	0.010	5.95E-08	29.244
	rs604302	C	T	-0.058	0.012	3.07E-06	21.653
	rs6920211	C	T	0.054	0.012	3.14E-06	21.805
	**Ferritin**						
	rs1050045	C	T	-0.043	0.009	4.12E-06	21.023
	rs12693541	T	C	-0.106	0.014	4.18E-14	57.327
	rs16976620	A	G	-0.081	0.016	4.52E-07	25.374
	rs173780	A	G	-0.068	0.015	3.35E-06	21.629
	rs1752162	T	C	0.061	0.013	4.80E-06	21.036
	rs2413450	C	T	0.056	0.010	3.57E-09	34.624
	rs368243	C	T	-0.051	0.009	3.80E-08	30.309
	rs4376025	T	C	0.046	0.010	3.62E-06	21.437
	rs7395347	T	C	0.045	0.010	3.20E-06	21.714
	rs7603193	T	C	-0.129	0.027	1.38E-06	23.343
	rs9322487	A	G	0.085	0.018	3.47E-06	21.473
	**Transferrin Saturation**						
	rs11046313	A	C	-0.056	0.011	6.86E-07	24.647

	rs2061336	G	A	0.093	0.018	1.99E-07	26.936
	rs2841000	T	C	-0.051	0.011	2.78E-06	22.038
	rs4790859	G	A	-0.048	0.010	2.29E-06	22.423
	rs604302	C	T	-0.058	0.012	3.26E-06	21.803
	rs8177272	A	G	-0.097	0.011	5.52E-20	83.740
	rs855791	G	A	0.192	0.010	3.50E-80	361.753
	rs9389269	C	T	0.055	0.011	9.78E-07	24.028
	rs9990333	T	C	0.049	0.010	7.37E-07	24.298
	**Transferrin**						
	rs10055024	T	C	0.051	0.010	8.98E-07	24.331
	rs11680788	T	C	-0.115	0.025	4.57E-06	21.028
	rs12371237	A	C	-0.050	0.011	3.48E-06	21.662
	rs1495741	A	G	0.083	0.012	1.57E-11	45.729
	rs17376530	T	C	-0.188	0.017	5.43E-30	129.960
	rs1865383	T	G	-0.052	0.011	2.17E-06	22.347
	rs3811658	T	C	0.388	0.011	1.00E-200	1269.059
	rs4291160	G	T	0.055	0.012	3.68E-06	21.567
	rs6486121	T	C	-0.056	0.011	1.04E-07	28.546
	rs7616492	A	G	-0.049	0.011	4.02E-06	21.336

Table S1. Characteristics of SNPs used as instrumental variables for serum iron status in MR analysis.

Table S2. The removed instrumental variables for serum iron status in MR analysis.

Table S3. The Cochran's Q test, Egger intercept test and MR-PRESSO of iron status on retinal degenerative diseases in the MR analysis.

Figure S1. Scatter plots of genetic correlations of serum iron status and retinal degenerative diseases using different MR methods.

Figure S2. Leave-one-out analysis of the causal relationship between serum iron status and retinal degenerative diseases.

Figure S3. Funnel plots of the causal relationship between serum iron status and retinal degenerative diseases.

Table S1. Characteristics of SNPs used as instrumental variables for serum iron status in MR analysis.

MR, mendelian randomization, SNP, single nucleotide polymorphism; β, the per-allelic effect on the level of serum iron, log10 ferritin, transferrin, and transferrin saturation; F statistic, the strength of the SNP for the corresponding exposure.

Table S2. The removed instrumental variables for serum iron status in MR analysis.

Disease	SNP	effect_ allele	other_ allele	β	SE	*p*-value	Reason
**AMD (whether dry or wet)**	rs2645490	A	G	-0.0496	0.0108	4.14E-06	Failed the Mendelian hypothesis test
	rs6920211	C	T	0.0537	0.0115	3.14E-06	Confounding factors
	rs1800562	A	G	0.3724	0.02	3.96E-77	Confounding factors
	rs651007	T	C	-0.0597	0.0116	2.54E-07	Confounding factors
	rs806970	T	C	-0.0977	0.0197	6.93E-07	Confounding factors
	rs9389269	C	T	0.0549	0.0112	9.78E-07	Confounding factors
	rs1110446	T	C	0.0563	0.0122	4.18E-06	Confounding factors
	rs1495741	A	G	0.0825	0.0122	1.57E-11	Confounding factors

**Wet AMD**	rs4376025	T	C	0.0463	0.01	3.62E-06	Failed the Mendelian hypothesis test
	rs173780	A	G	-0.0679	0.0146	3.35E-06	Failed the Mendelian hypothesis test
	rs1752162	T	C	0.061	0.0133	4.80E-06	Failed the Mendelian hypothesis test
	rs16976620	A	G	-0.0811	0.0161	4.52E-07	Failed the Mendelian hypothesis test
	rs2645490	A	G	-0.0496	0.0108	4.14E-06	Failed the Mendelian hypothesis test
	rs2413450	C	T	0.0559	0.0095	3.57E-09	Failed the Mendelian hypothesis test
	rs6920211	C	T	0.0537	0.0115	3.14E-06	Confounding factors
	rs1800562	A	G	0.3724	0.02	3.96E-77	Confounding factors
	rs651007	T	C	-0.0597	0.0116	2.54E-07	Confounding factors
	rs9389269	C	T	0.0549	0.0112	9.78E-07	Confounding factors
	rs1110446	T	C	0.0563	0.0122	4.18E-06	Confounding factors
	rs1495741	A	G	0.0825	0.0122	1.57E-11	Confounding factors
**Dry AMD**	rs2645490	A	G	-0.0496	0.0108	4.14E-06	Failed the Mendelian hypothesis test
	rs6920211	C	T	0.0537	0.0115	3.14E-06	Confounding factors
	rs1800562	A	G	0.3724	0.02	3.96E-77	Confounding factors
	rs651007	T	C	-0.0597	0.0116	2.54E-07	Confounding factors
	rs806970	T	C	-0.0977	0.0197	6.93E-07	Confounding factors
	rs9389269	C	T	0.0549	0.0112	9.78E-07	Confounding factors
	rs1110446	T	C	0.0563	0.0122	4.18E-06	Confounding factors
	rs1495741	A	G	0.0825	0.0122	1.57E-11	Confounding factors
**Diabetic Retinopathy**	rs2645490	A	G	-0.0496	0.0108	4.14E-06	Failed the Mendelian hypothesis test
	rs8177272	A	G	-0.097	0.0106	5.52E-20	Failed the Mendelian hypothesis test
	rs9990333	T	C	0.0488	0.0099	7.37E-07	Failed the Mendelian hypothesis test for part of the iron status
	rs806970	T	C	-0.0977	0.0197	6.93E-07	Failed the Mendelian hypothesis test
	rs2235233	C	T	-0.069	0.0129	8.81E-08	Failed the Mendelian hypothesis test
	rs221834	G	C	0.1226	0.0205	2.38E-09	Failed the Mendelian hypothesis test
	rs4790859	G	A	-0.0483	0.0102	2.29E-06	Failed the Mendelian hypothesis test
	rs855791	G	A	0.1921	0.0101	3.50E-80	Failed the Mendelian hypothesis test for part of the iron status
	rs3811658	T	C	0.3883	0.0109	1.00E-200	Failed the Mendelian hypothesis test
	rs9268633	G	A	0.0717	0.0128	2.31E-08	Failed the Mendelian hypothesis test
	rs1800562	A	G	0.3724	0.02	3.96E-77	Confounding factors
	rs651007	T	C	-0.0597	0.0116	2.54E-07	Confounding factors
	rs946526	C	T	0.122	0.0261	2.98E-06	Confounding factors
	rs1110446	T	C	0.0563	0.0122	4.18E-06	Confounding factors

	rs174577	A	C	0.0684	0.0107	1.90E-10	Confounding factors
**Proliferative Diabetic Retinopathy**	rs1033478	C	T	-0.0551	0.0108	3.63E-07	Failed the Mendelian hypothesis test
	rs2075672	G	A	0.0557	0.0103	5.95E-08	Failed the Mendelian hypothesis test
	rs7209063	G	C	-0.0483	0.0104	3.61E-06	Failed the Mendelian hypothesis test
	rs2645490	A	G	-0.0496	0.0108	4.14E-06	Failed the Mendelian hypothesis test
	rs8177272	A	G	-0.097	0.0106	5.52E-20	Failed the Mendelian hypothesis test
	rs2235233	C	T	-0.069	0.0129	8.81E-08	Failed the Mendelian hypothesis test
	rs9389269	C	T	0.0549	0.0112	9.78E-07	Failed the Mendelian hypothesis test
	rs221834	G	C	0.1226	0.0205	2.38E-09	Failed the Mendelian hypothesis test
	rs604302	C	T	-0.0579	0.0124	3.26E-06	Failed the Mendelian hypothesis test for part of the iron status
	rs4790859	G	A	-0.0483	0.0102	2.29E-06	Failed the Mendelian hypothesis test
	rs855791	G	A	0.1921	0.0101	3.50E-80	Failed the Mendelian hypothesis test for part of the iron status
	rs744653	T	C	0.0916	0.0144	2.00E-10	Failed the Mendelian hypothesis test
	rs17376530	T	C	-0.1881	0.0165	5.43E-30	Failed the Mendelian hypothesis test
	rs3811658	T	C	0.3883	0.0109	1.00E-200	Failed the Mendelian hypothesis test
	rs9990333	T	C	-0.067	0.0101	3.01E-11	Failed the Mendelian hypothesis test for part of the iron status
	rs10055024	T	C	0.0513	0.0104	8.98E-07	Failed the Mendelian hypothesis test
	rs9268633	G	A	0.0717	0.0128	2.31E-08	Failed the Mendelian hypothesis test
	rs4291160	G	T	0.0548	0.0118	3.68E-06	Failed the Mendelian hypothesis test
	rs1495741	A	G	0.0825	0.0122	1.57E-11	Failed the Mendelian hypothesis test
	rs1354342	G	A	-0.1264	0.0239	1.28E-07	Failed the Mendelian hypothesis test
	rs2165554	T	C	-0.0483	0.0105	4.19E-06	Failed the Mendelian hypothesis test
	rs2374503	C	G	-0.0482	0.0103	2.95E-06	Failed the Mendelian hypothesis test
	rs1800562	A	G	0.3724	0.02	3.96E-77	Confounding factors
	rs651007	T	C	-0.0597	0.0116	2.54E-07	Confounding factors
	rs806970	T	C	-0.0977	0.0197	6.93E-07	Confounding factors
	rs946526	C	T	0.122	0.0261	2.98E-06	Confounding factors
	rs1110446	T	C	0.0563	0.0122	4.18E-06	Confounding factors
	rs174577	A	C	0.0684	0.0107	1.90E-10	Confounding factors

**Nonproliferative Diabetic Retinopathy**	rs12731	A	G	0.0473	0.01	2.52E-06	Failed the Mendelian hypothesis test
	rs7172337	C	T	0.0523	0.0111	2.63E-06	Failed the Mendelian hypothesis test
	rs7209063	G	C	-0.0483	0.0104	3.61E-06	Failed the Mendelian hypothesis test
	rs13038647	C	T	0.0463	0.01	3.80E-06	Failed the Mendelian hypothesis test
	rs855791	G	A	0.1868	0.0101	4.31E-77	Failed the Mendelian hypothesis test for part of the iron status
	rs2645490	A	G	-0.0496	0.0108	4.14E-06	Failed the Mendelian hypothesis test
	rs744653	T	C	0.0916	0.0144	2.00E-10	Failed the Mendelian hypothesis test
	rs10935059	T	G	-0.1568	0.033	2.02E-06	Failed the Mendelian hypothesis test
	rs9990333	T	C	-0.067	0.0101	3.01E-11	Failed the Mendelian hypothesis test for part of the iron status
	rs9268633	G	A	0.0717	0.0128	2.31E-08	Failed the Mendelian hypothesis test
	rs1354342	G	A	-0.1264	0.0239	1.28E-07	Failed the Mendelian hypothesis test
	rs2165554	T	C	-0.0483	0.0105	4.19E-06	Failed the Mendelian hypothesis test
	rs2374503	C	G	-0.0482	0.0103	2.95E-06	Failed the Mendelian hypothesis test
	rs12978009	A	G	-0.0642	0.0138	3.17E-06	Failed the Mendelian hypothesis test
	rs2275901	A	G	0.0601	0.0126	1.77E-06	Failed the Mendelian hypothesis test
	rs1800562	A	G	0.3724	0.02	3.96E-77	Confounding factors
	rs651007	T	C	-0.0597	0.0116	2.54E-07	Confounding factors
	rs806970	T	C	-0.0977	0.0197	6.93E-07	Confounding factors
	rs2235233	C	T	-0.069	0.0129	8.81E-08	Confounding factors
	rs946526	C	T	0.122	0.0261	2.98E-06	Confounding factors
	rs1110446	T	C	0.0563	0.0122	4.18E-06	Confounding factors
	rs174577	A	C	0.0684	0.0107	1.90E-10	Confounding factors

Table S3. The Cochran's Q test, Egger intercept test and MR-PRESSO of iron status on retinal degenerative diseases in the MR analysis.

**Exposure**	**Outcome**	**Cochran's Q test**				**Egger intercept test**		**MR-PRESSO**	
		**Q-IVW**	***p*-value**	**MR Egger**	***p*-value**	**Egger-intercept**	***p*-value**	**RSSobs**	***p*-value of global test**
**Serum Iron**	**AMD (whether dry or wet)**	5.521	0.597	4.060	0.669	0.022	0.272	10.870	0.521
**Ferritin**		9.580	0.478	9.451	0.397	0.010	0.734	11.166	0.521

**Transferrin Saturation**		18.501	0.018	18.257	0.011	0.009	0.769	21.292	0.064
**Serum Iron**	**Wet AMD**	5.721	0.573	5.716	0.456	0.002	0.943	6.417	0.707
**Transferrin Saturation**		22.651	0.204	20.675	0.241	0.018	0.220	26.601	0.309
**Serum Iron**	**Dry AMD**	6.153	0.522	5.879	0.437	0.115	0.620	7.505	0.632
**Ferritin**		5.086	0.885	4.977	0.836	-0.012	0.749	6.059	0.898
**Transferrin Saturation**		17.823	0.023	17.779	0.013	-0.005	0.899	19.844	0.085
**Transferrin**		18.429	0.428	17.550	0.418	0.011	0.369	23.776	0.438
**Ferritin**	**Retinitis Pigmentosa**	5.360	0.866	4.145	0.902	-0.110	0.299	6.666	0.868
**Transferrin**		7.896	0.969	7.893	0.952	-0.002	0.959	8.650	0.972
**Serum Iron**	**Diabetic Retinopathy**	4.551	0.804	3.336	0.852	0.010	0.307	8.586	0.681
**Ferritin**		12.039	0.282	11.898	0.219	-0.006	0.752	15.014	0.278
**Transferrin Saturation**		2.530	0.639	1.744	0.627	-0.036	0.441	3.839	0.685
**Transferrin**		8.593	0.898	5.297	0.981	0.0162	0.091	10.812	0.862
**Serum Iron**	**Proliferative Diabetic Retinopathy**	1.527	0.958	1.338	0.931	-0.005	0.682	1.904	0.971
**Ferritin**		7.118	0.714	7.098	0.627	-0.003	0.889	8.676	0.710
**Transferrin Saturation**		0.329	0.954	0.286	0.967	-0.010	0.855	0.570	0.956
**Transferrin**		2.232	0.946	0.993	0.986	0.020	0.308	2.916	0.952
**Serum Iron**	**Nonproliferative Diabetic Retinopathy retinopathy**	0.958	0.916	0.810	0.847	-0.102	0.726	1.576	0.914
**Ferritin**		8.341	0.596	8.328	0.501	-0.009	0.911	10.256	0.603
**Transferrin Saturation**		11.890	0.156	6.199	0.517	0.116	0.048	17.912	0.213
**Transferrin**		3.983	0.913	3.897	0.866	0.010	0.777	4.207	0.952

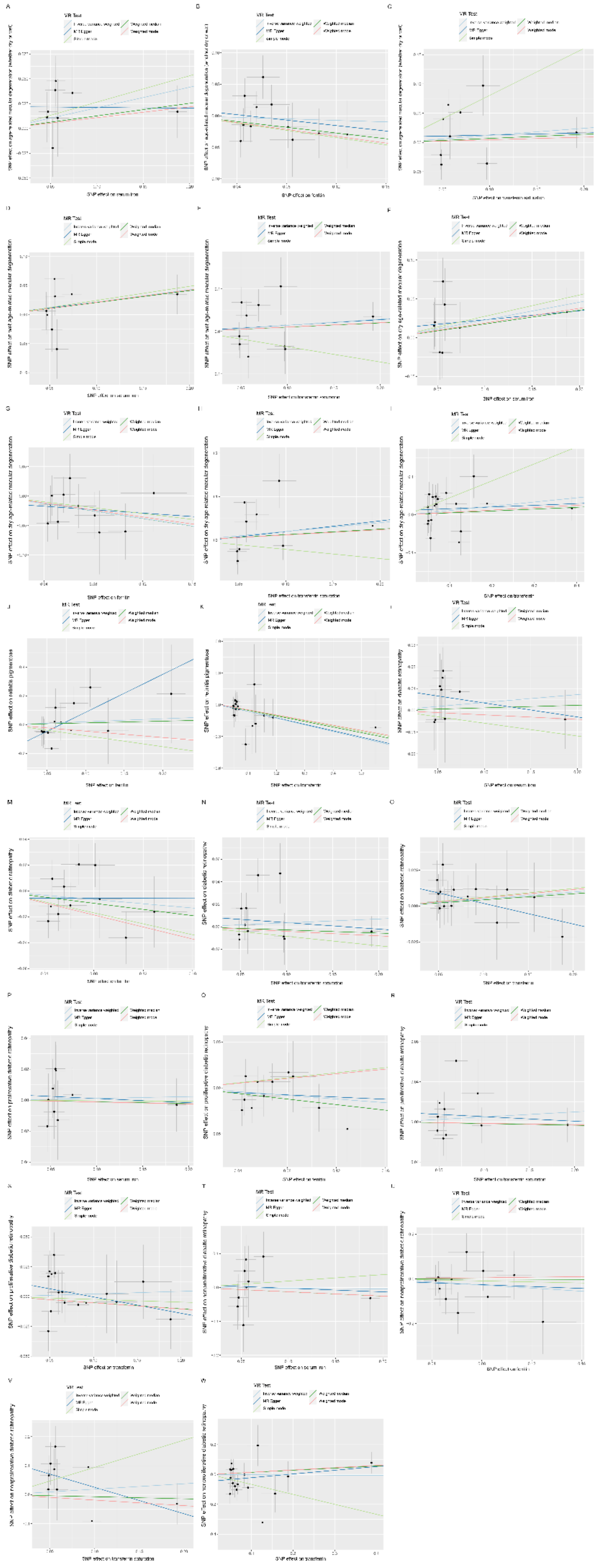
A
B
C
D
E
F
G
H
I
J
K
L
M
N
O
P
Q
R
S
T
U
V
W
MR Test
Inverse variance weighted
MR Egger
Simple mode
Weighted median
Weighted mode

Figure S1. Scatter plots illustrating the associations between different serum iron status markers and retinal degenerative diseases. The five methods used in this study are depicted including IVW, MR Egger, Simple model, Weighted median, and Weight mode. The vertical axis in scatter plots represents the effect of selected SNPs on the outcome, the horizontal axis is the effect of selected SNPs on the exposure, and each distinct point represents an instrumental SNP. SNP, single-nucleotide polymorphisms; MR, Mendelian randomization.

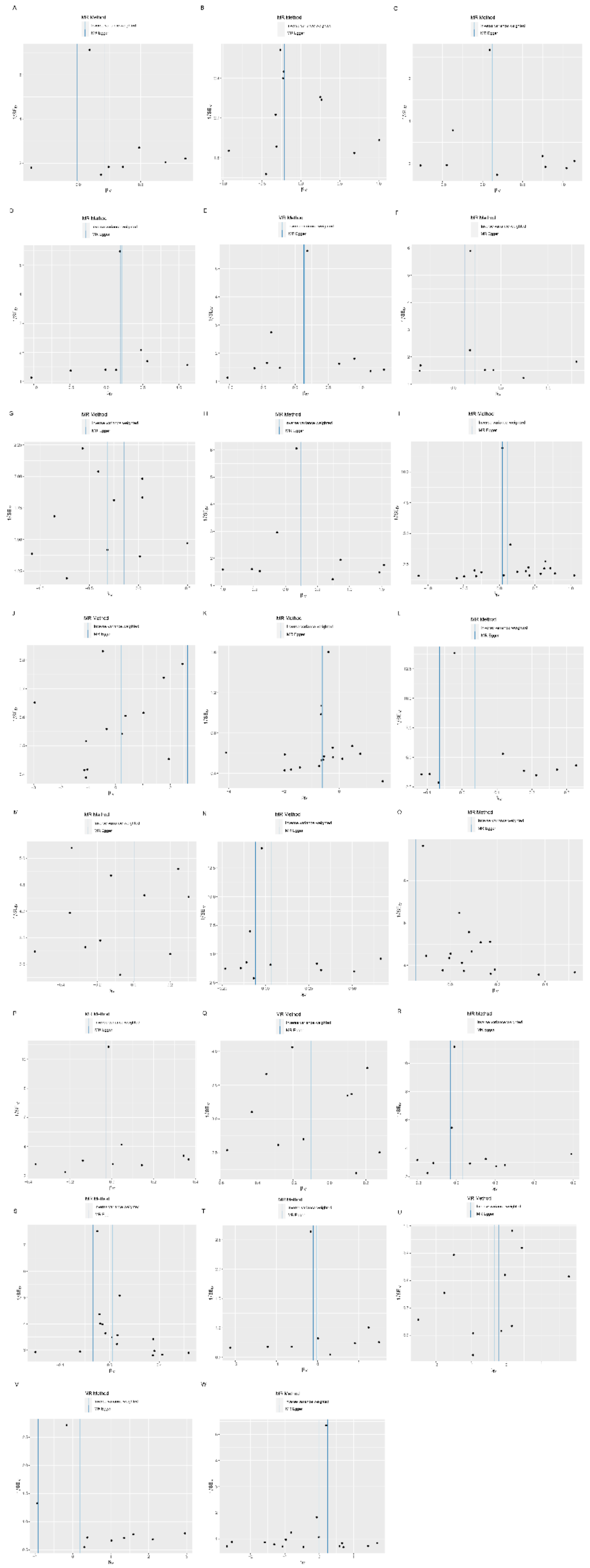

Figure S2. Funnel plots employed to evaluate the causal association between different serum iron status markers and retinal degenerative diseases in MR analysis. MR, Mendelian randomization; IV, instrumental variable; SE, standard error.

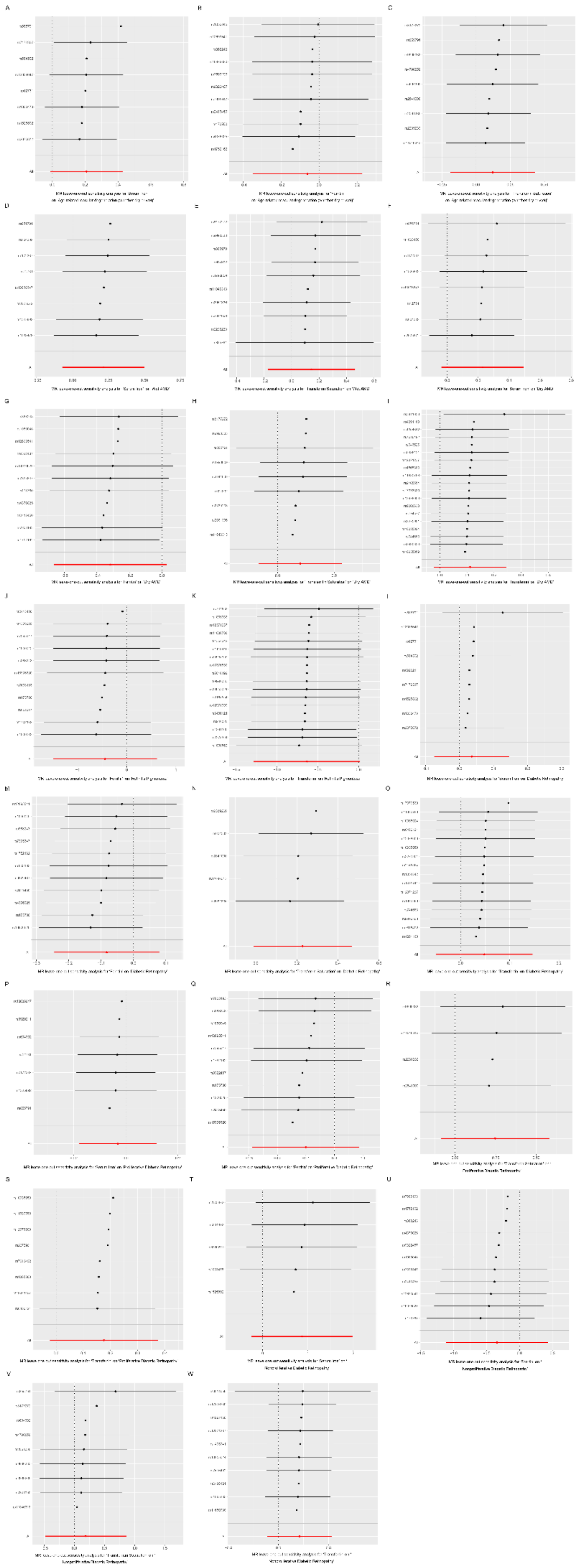

Figure S3. Leave-one-out analysis of in the causal relationship between different serum iron status markers and retinal degenerative diseases. MR, Mendelian randomization.
